# Public preferences for investigative genetic genealogy (IGG) programs: A discrete choice experiment

**DOI:** 10.1016/j.gimo.2026.104499

**Published:** 2026-09-30

**Authors:** Nijole P. Tjader, Natalie Riva Smith, Nicola B. Campoamor, Amy L. McGuire, Christi J. Guerrini, John F.P. Bridges, Norah L. Crossnohere

**Affiliations:** 1 Department of Biomedical Informatics, College of Medicine, The Ohio State University Wexner Medical Center, Columbus, OH; 2 Department of Health Policy and Management, University of Pittsburgh, Pittsburgh, PA; 3 Center for Medical Ethics and Health Policy, Baylor College of Medicine, Houston, TX; 4 Department of Internal Medicine, Division of General Internal Medicine, College of Medicine, The Ohio State University Wexner Medical Center, Columbus, OH

**Keywords:** Ethical, legal, and social implications, Genetic privacy, Law enforcement, Preference

## Abstract

**Purpose:**

Investigative genetic genealogy (IGG) uses data from genetic relative-finder services to assist law enforcement in identifying unknown individuals through crime scene DNA. Its use raises concerns about privacy infringements and acceptable trade-offs for public safety. We assessed the public acceptability of IGG and preferences for specific IGG programs.

**Methods:**

We conducted a survey with an embedded choice experiment among US adults recruited through a probability-based panel. IGG acceptability was assessed through attitudinal questions. Preferences for IGG programs were assessed using a discrete choice experiment with 6 attributes: crime type, additional cases solved, customer privacy, privacy of genetic relatives, case status, and priority populations. Choice data were analyzed using conditional logistic regression to generate relative attribute importance.

**Results:**

Among 1123 participants (52% female, 61% White), 88% believed it was ever acceptable to use IGG to investigate any crime, with only 5% indicating it should never be used. Privacy of genetic relatives and additional cases solved were the most influential factors in the selection of IGG programs (relative attribute importance, 33% and 31%, respectively).

**Conclusion:**

IGG was generally acceptable to US adults. Policymakers and experts developing IGG programs may improve public trust by ensuring that the programs are maximally effective and by embedding privacy protections for noncustomer genetic relatives.

## Introduction

Participation in genetic relative-finder services has grown rapidly because of the development of inexpensive and readily available direct-to-consumer DNA testing kits.^1^ An estimated 53 million individuals worldwide have allowed the sharing of their DNA data in databases from 5 major direct-to-consumer testing companies as of 2025.^2^ Individuals agree to share their DNA data with the intention of learning about their genetic ancestry and heritable medical traits, as well as identifying unknown genetic relatives. These genetic relative-finder services enable genetic genealogy, defined as identifying one’s genetic relatives based on shared DNA.^3^

Investigative genetic genealogy (IGG) is the practice of uploading crime scene DNA to genetic genealogy databases with the intention of identifying the unknown person’s genetic relatives and locating the person in a family tree.^4^, ^5^, ^6^, ^7^, ^8^, ^9^ Investigative genetic genealogy has been used to assist law enforcement with identifying perpetrators of violent crimes in the United States and internationally,^9^^,^^10^ including to identify the Golden State Killer^11^ and the perpetrator of the Idaho college murders.^12^ It can also be used to identify unidentified human remains^9^^,^^13^ and exonerate wrongly convicted individuals.^14^ According to the Forensic Genetic Genealogy Project database, which tracks cases cleared globally using IGG, over 1600 cases of criminal offenses or unidentified human remains have been solved by IGG and publicized as of 2025.^10^^,^^15^ IGG is now well integrated into many US law enforcement agencies, and some, such as the Federal Bureau of Investigation, have established programs with stable funding and specialized genetic genealogy practitioners.

The expanding use of IGG has raised ethical, legal, and social concerns about key aspects of the practice.^10^^,^^15^ IGG programs must be implemented with consideration for issues such as the privacy and security of genetic data,^16^, ^17^, ^18^, ^19^ the informed consent of genetic genealogy database participants and their relatives,^18^^,^^19^ the overrepresentation of samples with Northern and Western European ancestry,^20^ and the potential for misuse.^21^ As such, there is ongoing debate about how to maximize the benefits of IGG to society while also reducing possible harms. Professional and academic bodies are working to balance the public benefit of IGG with consideration of ethical concerns and are calling for the establishment of standards and certifications for the use of IGG.^6^^,^^19^^,^^22^

Given the trade-offs between personal privacy and societal benefit inherent to IGG, an understanding of how the public values aspects of IGG programs is useful for informing the design of future IGG programs. Prior studies have indicated that members of the public generally support the use of IGG to solve violent crimes^23^, ^24^, ^25^, ^26^, ^27^ but have not examined support for specific practices of IGG or how the public is willing to make trade-offs among these practices. We assess the public acceptability of IGG and identify preferred characteristics of IGG programs among members of the US public. Findings from this research may inform policy about IGG by elucidating current endorsement of the practice among the US public and identifying characteristics of programs that may further improve IGG acceptability.

## Materials and Methods

This survey measured the acceptability of IGG and used a discrete choice experiment (DCE) to assess the relative importance of IGG program attributes that vary in terms of privacy protections and social benefit. A DCE is a method to elicit preferences by asking respondents to choose among hypothetical alternatives that are described by multiple attributes with varying levels, allowing estimation of the relative importance of those attributes and preference among the levels.^28^ Our DCE followed good practices for choice experiment design, conduct, and analysis.^29^^,^^30^ It was developed from a literature review, expert interviews,^8^ and focus groups with members of the US general public^31^ and revised following a rigorous pretesting process.^32^ All activities of the study were approved by the Baylor College of Medicine Institutional Review Board (H-47654). We followed the Discrete Choice Experiment Reporting Checklist reporting guidelines.^33^

### Participants

The survey was administered to members of the AmeriSpeak Probability-Based Panel maintained by the National Opinion Research Center.^34^ Individuals were required to be aged at least 18 years to participate. The survey was designed to enroll individuals with nationally representative demographics in Pilot 1 (*n* = 63) and in the Main study group (*n* = 1019), while Pilot 2 (*n* = 41) included only non-White individuals by self-reported race. The first page of the survey provided brief information about the survey and indicated that continuing with the survey implied consent. Participants received approximately $3 worth of AmeriPoints for completing the survey.

### Survey instrument

The survey provided brief informational material to introduce respondents to concepts related to genetics, genealogy, and IGG, followed by an interactive knowledge check to confirm their understanding. The survey included a DCE to assess preferences regarding IGG programs and closed-ended questions that assessed the acceptability of IGG and participant characteristics.

The DCE to assess preferences began with an introduction and an example choice task (Figure 1). Participants were presented with choice tasks that included 2 unlabeled IGG programs and an opt-out in which IGG should not be used at all and were asked to choose which option was better for society. The DCE featured 6 attributes of potential IGG programs and 3 associated levels for each. The attributes included crimes that IGG could help solve, such as violent or nonviolent crime (crime type); the number of additional cases IGG could help solve (additional cases solved); the privacy of genetic genealogy database customers (customer privacy); the privacy of relatives who are not genetic genealogy customers (privacy of genetic relatives); when to use IGG during an investigation relative to other police methods (case status); and whether to prioritize cases involving different groups of people (priority populations) (Table 1). As noted in the survey, attributes and levels were a combination of current practices in IGG and practices or outcomes that are not currently implemented or possible. The combinations were selected because they were identified by the public as issues of concern or interest during the survey design process. In addition, levels were selected to encourage trade-offs, consistent with DCE best practices. The DCE used an Ngene-generated D-efficient main-effects experimental design given no hypothesized interaction between attributes (ChoiceMetrics).^35^ Participants completed 9 choice tasks from 1 of 3 randomly assigned blocks, with task order randomized.

**Figure 1:**
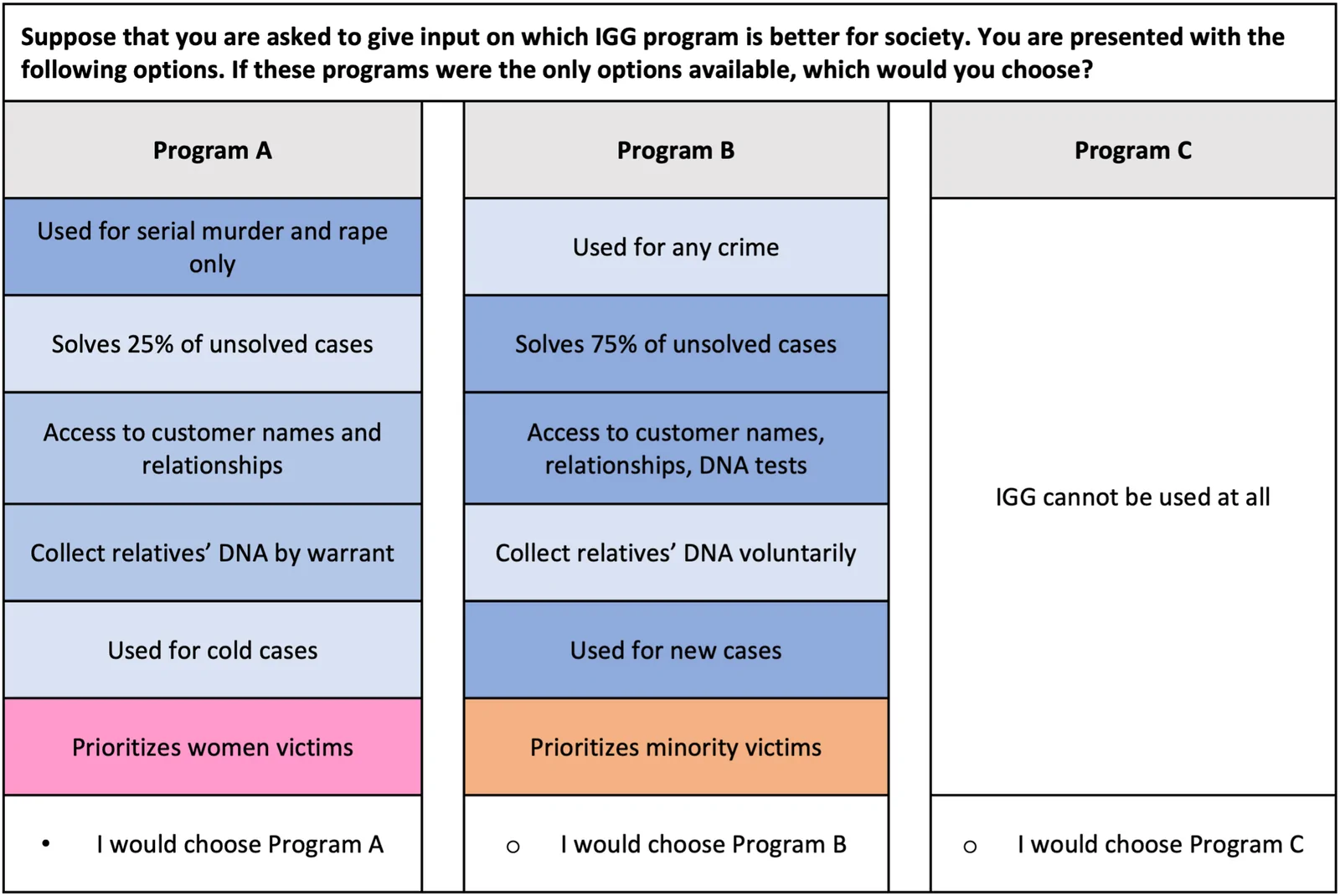
**Example discrete choice experiment task.** IGG, investigative genetic genealogy. (Republished with permission from Campoamor et al^32^*The Patient*.).

**Table 1:** IGG attributes and levels in DCE

Attribute	Description^a^	Levels
Privacy of genetic relatives	During the process of IGG, police may obtain DNA from a genetic relative to narrow the investigation. Limits can be placed on how the police get DNA from relatives.	• Collect DNA voluntarily • Collect DNA by warrant • Collect DNA secretly
Additional cases solved	Some crimes cannot be solved using standard police methods but can be solved using IGG.	• 75% more cases • 50% more cases • 25% more cases
Priority populations	IGG programs may receive additional funding to address past injustices. This additional funding can be used to prioritize cases involving different groups of people.	• Women • Minorities • Wrongly convicted
Crime type	IGG can be used to investigate different types of crime. Given concerns that IGG might intrude on some people’s privacy, IGG programs may limit the types of crimes they are used to investigate.	• Serial murder/rape • Violent crime • Any crime
Case status	IGG is one of many methods the police might use in an investigation. Limits can be placed on when IGG can be used depending on the age and status of the case.	• Cold cases • Active cases • New cases
Customer privacy	When building the perpetrator's family tree, police submit perpetrator DNA to a genetic genealogy company, which searches its customer database for genetic relatives. IGG programs may limit what customer information police can access.	• Customer names • Customer names, relationships • Customer names, relationships, DNA test results

*DCE*, discrete choice experiment; *IGG*, investigative genetic genealogy.

a Adapted from survey. See full descriptions provided to respondents in Supplemental Materials.

Closed-ended questions throughout the survey evaluated IGG acceptability and participant attitudes and characteristics. Participants answered whether use of IGG would be acceptable for investigating crimes, identifying unidentified human remains, and identifying deceased, abandoned infants (5 response options ranging from always to never, as well as an option for not sure). They also answered whether IGG should be regulated and by whom. Additional closed-ended attitudinal questions introduced the 6 featured attributes of IGG programs and asked participants whether each attribute should be considered as part of the program limitations and priorities (yes/no). The final module of the survey asked about individuals’ lived experiences that might have influenced their choices in the experiment. This included their experiences with crime and justice, including whether they were victims or perpetrators of crimes, the perceived level of crime in their neighborhoods, perceptions of law enforcement, and whether they had ever worked in law enforcement or the legal field. Sociodemographic information about participants was provided directly by the National Opinion Research Center.

### Data analysis

We pooled responses from Pilot 1, Pilot 2, and the Main study group for analysis. The DCE was modeled using conditional logistic regression with adjustment for observed heteroscedasticity across the main survey and pilot surveys.^36^ In the primary model, the dependent variable was the choice of IGG program, and the independent variables were the attribute levels. Effects coding of independent variables was used to allow estimation of preference coefficients for all levels of each attribute, including the reference level.^37^ We used the main effects from this model to calculate relative attribute importance (RAI), an indication of how each attribute contributes to preferences relative to other attributes.^38^ The model also estimated interactions between participant demographics and the opt-out to assess potential differences in IGG program acceptability. *P* values for parameter coefficients were determined using Wald tests.

Participant demographics, experiences with crime and justice, participation in genetic genealogy, and IGG attitudinal questions were assessed using descriptive statistics. Responses to the attribute rating items were binary (yes/no). All analyses were conducted using Stata 16 (StataCorp).

## Results

### Participant demographics and background

A total of 1123 individuals completed the survey and were included in the pooled data analysis. The mean participant age was 49 years (range 18-90) (Table 2), and 52% identified as female. Most respondents (62%) had less than a bachelor’s degree. A small proportion reported work experience in law enforcement (4%) or the legal field (5%). Eighteen percent of individuals had previously participated in genetic genealogy; of those who had not, 29% were interested in participating, while 43% were not interested, and 28% were not sure. Thirty-four percent of individuals had heard of IGG before taking the survey. Additional demographics for participants in Pilot 1, Pilot 2, and the Main study group are described (Supplemental Table 1).

**Table 2:** Patient demographics

Characteristic	*n* = 1123	
Gender	Female	52%
Age	≥45	56%
Race and Ethnicity	Non-Hispanic White	61%
	Non-Hispanic Black	13%
	Hispanic	18%
	Asian-Pacific Islander^a^	4%
	Other, 2+^a^	5%
Education	High school or less	21%
	Some college	41%
	Bachelor’s degree or more	38%
Has worked in law enforcement		4%
Has worked in legal field		5%
Has participated in genetic genealogy		18%

a Non-Hispanic.

### Attitudes toward IGG

Only 5% of individuals in the main study group selected that IGG should never be used to solve crimes, and 88% agreed that IGG should be used to investigate crimes, always (20%), usually (22%), sometimes (37%), or rarely (9%); 8% selected that they were not sure (Supplemental Table 2). Individuals who had participated in genetic genealogy selected that IGG should never be used to solve crimes less often than those who had not participated (2% vs 5%, *P* = .04). Support was higher for IGG in the main study group to identify the deceased, with 95% agreeing that IGG should always (50%), usually (28%), sometimes (14%), or rarely (3%) be used to identify unidentified deceased individuals, compared with never (1%) or not sure (5%). Similarly, 92% agreed that it should always (55%), usually (22%), sometimes (12%), or rarely (3%) be used to identify deceased, abandoned infants, compared with never (2%) or not sure (6%). Most participants in the combined surveys agreed that IGG should be regulated (78%), with only 4% opposing any kind of regulation and 18% not sure. Among those who did not oppose regulation of IGG (96% answering “Yes” or “Not sure” combined, *n* = 1080), participants identified multiple potential regulatory bodies, including the federal government (54%), state governments (40%), county or city governments (21%), and police leadership (14%). When asked who should build family trees for IGG, 24% of the main study group selected the police, 67% selected professional or independent genetic genealogists, 23% selected other people who work in law enforcement, and 19% indicated they were not sure.

As assessed through attitudinal questions, individuals agreed that policies regarding IGG should protect customer privacy (79%), as well as the privacy of genetic relatives (82%) (Supplemental Table 3). A majority also felt that IGG use should be limited based on type of crime (71%) and case status (68%). Additionally, 63% agreed that the number of additional cases that could be solved through IGG should factor into its permissibility, whereas less than half (47%) supported the idea that IGG programs should prioritize certain populations.

### Preferences for IGG programs

In the DCE, privacy of genetic relatives was the most influential attribute on the choice of an IGG program (33% of the total RAI), followed by additional cases solved (31%), priority populations (15%), crime type (12%), and case status (8%) (Figure 2A). Customer privacy had a minimal impact on program choice, contributing just 1% to the total RAI.

**Figure 2:**
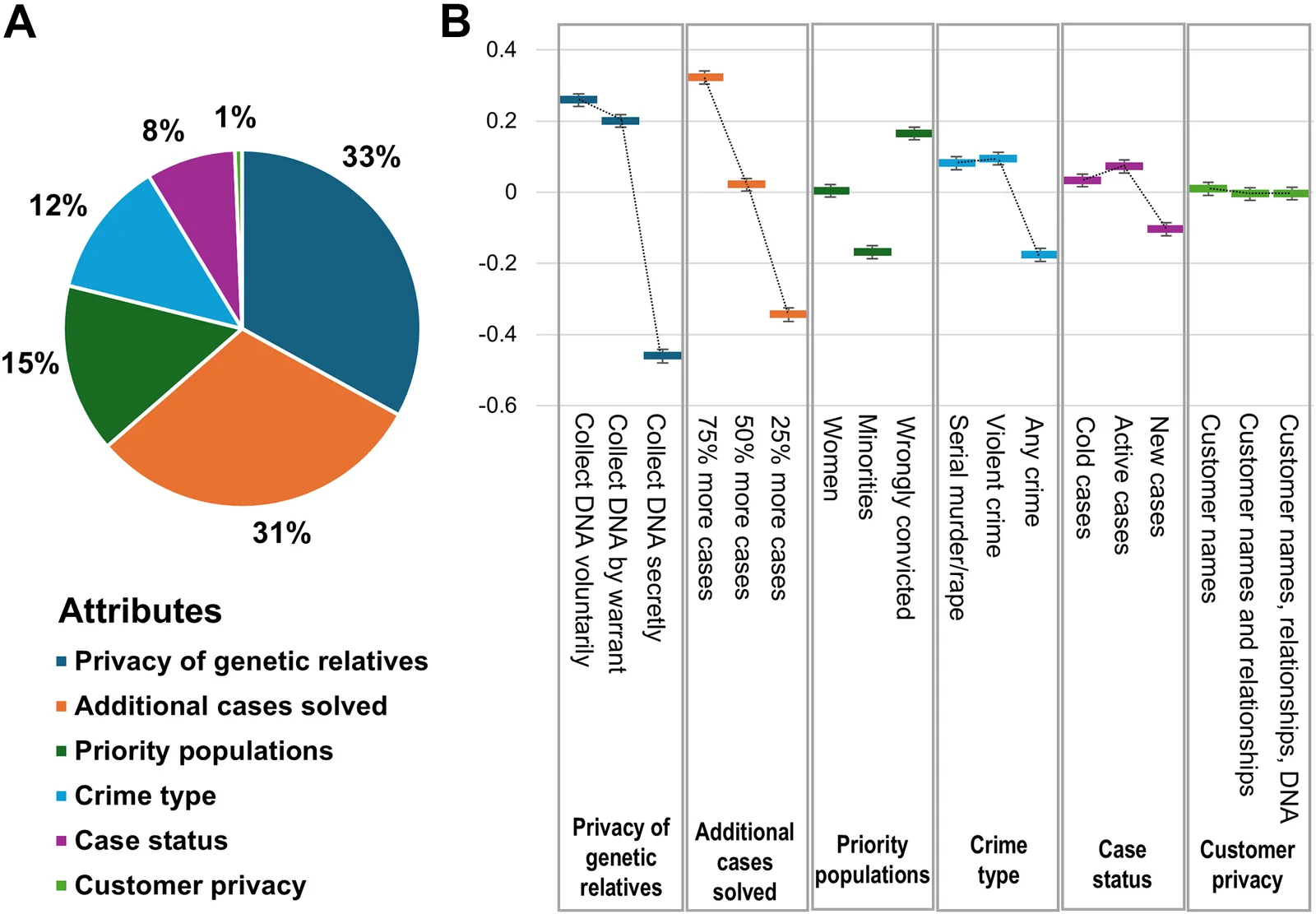
**Public preferences for investigative genetic genealogy program attributes.** A. Relative attribute importance. B. Beta utility value of the corresponding levels, connected for levels that are ordinal. Error bars indicate the SE.

The DCE also identified public preferences among levels of attributes (Table 3). For privacy of genetic relatives, participants preferred that DNA be collected voluntarily or by warrant and opposed secret collection of DNA (*P* < .01) (Figure 2B). When considering type of crime, participants preferred IGG programs to be used for violent crimes (*P* < .01) or serial rape and murder (*P* < .01) and opposed using IGG for any crime (*P* < .01). Participants favored using IGG for active cases simultaneously with other police methods (*P* < .01) or cold cases that were not solved using other methods (*P* = .07) and opposed use for new cases before other methods had been attempted (*P* < .01). They also favored programs that prioritized exonerating wrongly convicted individuals (*P* < .01) over programs that prioritized crimes committed against racial and ethnic minorities (*P* < .01) or crimes against women (*P* = .83). Customer privacy had a negligible effect on choice of IGG programs, regardless of the degree of privacy offered.

**Table 3:** Model estimates for IGG program attributes and opt-in predictor

Attributes (Levels)		Coefficient (95% CI)	*P* Value
Privacy of genetic relatives			
	DNA voluntarily	0.26 (0.22-0.30)	<.01
	DNA by warrant	0.2 (0.17-0.24)	<.01
	DNA secretly	−0.46 (−0.5 to −0.42)	<.01
Additional cases solved			
	75% more cases	0.32 (0.29-0.36)	<.01
	50% more cases	0.02 (−0.013 to 0.056)	.23
	25% more cases	−0.34 (−0.38 to −0.31)	<.01
Priority populations			
	Women	0.004 (−0.032 to 0.039)	.83
	Minorities	−0.17 (−0.2 to −0.13)	<.01
	Wrongly convicted	0.17 (0.13-0.2)	<.01
Crime type			
	Serial murder/rape	0.082 (0.046-0.12)	<.01
	Violent crime	0.094 (0.059-0.13)	<.01
	Any crime	−0.17 (−0.21 to −0.14)	<.01
Case status			
	Cold cases	0.033 (−0.0029 to 0.068)	.07
	Active cases	0.072 (0.036-0.11)	<.01
	New cases	−0.10 (−0.14 to −0.07)	<.01
Customer privacy			
	Names	0.009 (−0.027 to 0.045)	.62
	Names, relationships	−0.005 (−0.04 to 0.03)	.78
	Names, relationships, DNA tests	−0.004 (−0.039 to 0.031)	.81
Opt-in			
	Overall	1.6 (1.3-1.8)	<.01
	Female gender	−0.62 (−0.77 to −0.48)	<.01
	Age ≥45 years	0.6 (0.46-0.74)	<.01
	Asian-Pacific Islander^a^	1.8 (1-2.5)	<.01
	Black^a^	−0.62 (−0.81 to −0.44)	<.01
	Hispanic, any race	0.13 (−0.061 to 0.33)	.18
	Other race^a^	0.38 (0.0042-0.77)	.05
	Residence in metro area	−0.05 (−0.25 to 0.15)	.62
	Educated past high school	0.011 (−0.16 to 0.18)	.90
	Worked in law enforcement	0.35 (−0.044 to 0.75)	.08
	Worked in legal field	−0.17 (−0.52 to 0.18)	.32
	Genetic genealogy participant	0.41 (0.2-0.62)	<.01

*IGG*, investigative genetic genealogy.

a Non-Hispanic.

Across all choice tasks, participants selected that IGG should not be used at all (“opted out”) 1% of the time. Several participant characteristics were associated with opting in to IGG (Table 2). Black Americans were less likely to opt in to the use of IGG (*P* < .01), and Asian-Pacific Islander Americans were more likely to opt in (*P* < .01) compared with other racial and ethnic groups. Women were less likely to opt in than men (*P* < .01). Experience working in law enforcement or the legal field was not associated with opting in to the use of IGG. Those who had participated in genetic genealogy were more likely to opt in to the use of IGG (*P* < .01).

## Discussion

There was a high degree of public support for IGG, consistent with previous research on this technique and other forensic genetic innovations.^24^, ^25^, ^26^, ^27^^,^^31^^,^^39^ Many US adults viewed IGG as an acceptable tool for criminal investigations, with few agreeing that IGG should never be used to solve crimes. At the same time, the majority supported regulation of IGG practices. Our findings highlight that individuals have clear preferences for how IGG should be implemented, particularly regarding protecting the privacy of noncustomer relatives by limiting intrusive practices such as surreptitious DNA collection to build family trees. These results suggest that while IGG is broadly acceptable, there is a path for its governance structures to be better aligned with public expectations for effectiveness and privacy protection.

Privacy of genetic relatives was the largest driver of decision making between possible IGG programs. Law enforcement can conduct third-party testing, which involves collecting and testing DNA samples from relatives who are nonsuspects. This third-party testing helps narrow their investigation, reducing the time and resources needed. Typically, third-party testers provide their DNA knowingly and with consent. However, in most jurisdictions law enforcement can collect and test DNA secretly and without a warrant, for example, from discarded trash that contains an individual’s DNA. Our results indicate that the public has concerns about using this practice on relatives of suspects. Currently, guidance from the US Department of Justice recommends surreptitious DNA collection from nonsuspect family members only in the event that voluntary collection would compromise an investigation.^5^^,^^40^ Utah has codified this recommendation into law.^41^ There are other possible policy solutions to lessen public concerns about surreptitious, warrantless DNA collection and testing of nonsuspects. For example, Maryland bans surreptitious DNA collection from nonsuspect family members who previously declined to provide DNA voluntarily.^42^^,^^43^

Additional cases solved was the second largest driver of decision making between IGG programs. We proposed different levels of cases solved for the choice experiment, though it is challenging to ascertain how many additional cases could be solved by IGG. The United States has backlogged unsolved cases with DNA evidence, and demand for DNA testing has increased over the past 2 decades, outpacing the work capacity of laboratories.^44^ An estimated 200,000 unsolved cases include sexual assault kits,^44^^,^^45^ approximately 25% to 50% of kits include DNA samples of sufficient quality to test, and 50% to 60% of tested samples are linked to known offenders.^44^^,^^46^ The remaining 40% to 50% of kits with tested DNA might be solvable through IGG, as well as part of the estimated 11,000 unidentified deceased individuals in the United States.^47^ To align with public values and contribute to reducing backlogs, IGG programs should be optimized to solve as many cases as possible. To help achieve this, US professional organizations have organized to develop best practices and promote proficiency among laboratory and genetic genealogy professionals. Such agencies include the Investigative Genetic Genealogy Accreditation Board, which establishes professional standards for proficiency in IGG, and the National Technology Validation and Implementation Collaborative, which provides guidance to crime laboratories and investigative agencies developing IGG programs.^48^^,^^49^

Participants preferred IGG programs that prioritize exonerating wrongly convicted individuals. IGG has been used to exonerate only a few individuals in the United States.^50^, ^51^, ^52^ There are many possible reasons for this, including a lack of funding.^50^ The finding that the public supports prioritizing exoneration cases for IGG is consistent with results from other empirical studies and provides additional support for efforts to increase access to and uptake of IGG by postconviction counsel in cases where there is DNA from a putative perpetrator and earlier testing was inconclusive, unsuccessful, or potentially flawed.^24^^,^^31^ No differences in preferences for IGG programs that solve crimes against women were observed in aggregate or in a post hoc analysis of men and women separately.

Participants favored the use of IGG to solve violent crime, showed little preference for restricting its use to only serial offenders, and opposed extending its use to solve any crime. These findings align with opinion polls that found Americans considered investigating violent crime to be the top priority of police efforts.^53^ Currently, the US Department of Justice restricts the use of IGG to investigating violent crimes and identifying deceased homicide victims, and the only 2 IGG databases that allow law enforcement participation have similar terms of service restrictions.^5^^,^^40^^,^^54^ The public supports maintaining this policy of limiting IGG use to investigating only violent crime.

Although participants endorsed customer privacy in attitudinal questions (79%), this attribute had little impact on IGG program choices in the DCE (1% RAI). This divergence likely reflects differences between what the public views as generally important and how people make relative trade-offs in head-to-head comparisons; while customer privacy mattered to participants, it mattered less than other program attributes when choices required prioritization. Currently, law enforcement is not given special access to genetic genealogy databases beyond any other user and cannot access others’ DNA files.^5^ Prior research suggests that although protecting customer privacy is desirable, many individuals perceive participation in genetic genealogy databases as implying consent to this potential use of their information.^31^ Further, participation in genetic genealogy databases is voluntary, which may be part of why privacy concerns for participants were not prioritized compared with the privacy of genetic relatives.

The pooled survey participant demographics were representative of the US population by race, ethnicity, age, education, and gender.^55^ Acceptability of IGG varied by demographics, as indicated by differences in selecting that IGG should not be used. This effect may be partially driven by demographic patterns in attitudes toward law enforcement. According to one study, non-Hispanic Black individuals are less likely to hold pro-police views than non-Hispanic Whites, males are slightly more likely to hold pro-police views than females, and older individuals are more likely to hold pro-police attitudes.^56^ We measured experiences with law enforcement in the survey, but the study is not powered to formally analyze these effects. Further studies are needed to interpret these demographic associations.

In addition to substantive findings about preferences for IGG programs, this survey also provides insight into the alignment between attitudinal evaluations of attributes and preferences for attributes based on DCE findings. When comparing the attitudinal responses about IGG program attributes and the corresponding RAIs identified in the DCE, some attributes were consistently favored with both methods, while other attributes showed divergence in preference depending on the method used. For instance, privacy of genetic relatives received the strongest support through both types of questions, while crime type and case status consistently showed mid-range support. In contrast, customer privacy was highly endorsed in its attitudinal question despite a low RAI, while additional cases solved ranked highly in RAI but received more moderate support in its attitudinal question. This suggests that the DCE method revealed information about preferences through trade-offs that were not apparent from attitudinal survey questions alone, providing a more nuanced perspective on how the US public views IGG program practices.

This study has several limitations. First, there are a number of additional attributes that could influence public acceptability of IGG. We did not measure public views on single-nucleotide variant vs short tandem repeat genotyping. There are concerns about privacy and misuse of heritable trait data through single-nucleotide variant genotyping used by genetic genealogy databases, but law enforcement does not have access to customer DNA files.^57^ Previous studies have also identified expert concerns about law enforcement violating terms of service of genetic genealogy websites.^50^ This is closely related to customer privacy, which was measured in the choice experiment. We also did not address accreditation of laboratories or licensing of the individuals performing IGG in the choice experiment. Additionally, as noted above, attributes and levels in this study may not reflect all aspects of IGG programs that will be implemented by individual jurisdictions or that are necessarily possible in the real world. For instance, the attribute of additional cases solved by an IGG program was presented as relative increases of 25%, 50%, and 75%, which are likely not achievable for most IGG programs even if their unsolved violent case backlogs are relatively small. These levels were selected to elicit meaningful trade-offs across the choice sets in a hypothetical context, and as with choice experiments generally, these findings are not intended to provide definitive estimates of real-world choices.

### Conclusion

This is the first DCE to evaluate public preferences for practices of IGG programs among the US public to our knowledge. We identified public acceptability of IGG and priorities for programs, including privacy of genetic relatives and additional cases solved. Incorporating these priorities into public policy may improve acceptability of IGG among the US public. These findings are also valuable for future ethical, legal, and social implications research of IGG.

## ORCID

Norah L. Crossnohere: http://orcid.org/0000-0002-2811-1330

## Conflict of Interest

Christi J. Guerrini is an invited guest of the Investigative Genetic Genealogy Working Group of the Scientific Working Group on DNA Analysis Methods and a member of the Advisory Board of the Investigative Genetic Genealogy Accreditation Board. FORENSEQ is a trademark of Verogen Inc and Qiagen NV. The ForenSeq Study is not affiliated with the FORENSEQ mark. All other authors report no conflicts of interest.

## Additional Information

Supplementary Full Survey Instrument
