# Supplementary material for: Public preferences for investigative genetic genealogy (IGG) programs: A discrete choice experiment
Source: Genet Med Open. 2026 Sep 30;4:104499. doi: 10.1016/j.gimo.2026.104499 (PMC13743379; doi:10.1016/j.gimo.2026.104499)
Supplement: Supplementary file 2 [file mmc1.docx]

**Supplemental Table 1: Extended demographics**

|  | | **Pilot 1 (n=63), n (%)** | **Pilot 2 (n=41), n (%)** | **Main (n=1,019), n (%)** | **Combined (n=1,123), n (%)** |
| --- | --- | --- | --- | --- | --- |
| Gender | *Female* | 42 (67%) | 29 (71%) | 508 (50%) | 589 (52%) |
| Age | *18-29* | 1 (2%) | 8 (20%) | 160 (16%) | 169 (15%) |
|  | *30-44* | 14 (22%) | 14 (34%) | 298 (29%) | 326 (29%) |
|  | *45-59* | 9 (14%) | 4 (10%) | 230 (23%) | 243 (22%) |
|  | *60+* | 39 (62%) | 15 (37%) | 331 (32%) | 385 (34%) |
| Race/Ethnicity | *White, non-Hispanic* | 55 (87%) | 0 (0%) | 633 (62%) | 688 (61%) |
|  | *Black, non-Hispanic* | 2 (3%) | 12 (29%) | 127 (12%) | 141 (13%) |
|  | *Other, non-Hispanic* | 2 (3%) | 2 (5%) | 13 (1%) | 17 (2%) |
|  | *Hispanic* | 2 (3%) | 17 (41%) | 178 (17%) | 197 (18%) |
|  | *2+, non-Hispanic* | 1 (2%) | 8 (20%) | 25 (2%) | 34 (3%) |
|  | *Asian-Pacific Islander, non-Hispanic* | 1 (2%) | 2 (5%) | 43 (4%) | 46 (4%) |
| Education | *High school or less* | 8 (13%) | 9 (22%) | 220 (22%) | 237 (21%) |
|  | *Some college / Associate’s degree* | 15 (24%) | 9 (22%) | 434 (43%) | 458 (41%) |
|  | *Bachelor’s degree or more* | 40 (63%) | 23 (56%) | 365 (36%) | 428 (38%) |
| Marital status | *Married* | 39 (62%) | 25 (61%) | 530 (52%) | 594 (53%) |
|  | *Widowed* | 4 (6%) | 0 (0%) | 36 (4%) | 40 (4%) |
|  | *Divorced* | 7 (11%) | 1 (2%) | 115 (11%) | 123 (11%) |
|  | *Separated* | 4 (6%) | 3 (7%) | 52 (5%) | 59 (5%) |
|  | *Never married* | 9 (14%) | 12 (29%) | 286 (28%) | 307 (27%) |
| Employment status | *Working* | 34 (54%) | 21 (51%) | 652 (64%) | 707 (63%) |
|  | *Not working* | 29 (46%) | 20 (49%) | 367 (36%) | 416 (37%) |
| Income | *Under $60,000* | 28 (44%) | 20 (49%) | 454 (45%) | 502 (45%) |
|  | *Over $60,000* | 35 (56%) | 21 (51%) | 565 (55%) | 621 (55%) |
| Region | *Northeast* | 12 (19%) | 7 (17%) | 146 (14%) | 165 (15%) |
|  | *Midwest* | 18 (29%) | 9 (22%) | 281 (28%) | 308 (27%) |
|  | *South* | 16 (25%) | 14 (34%) | 352 (35%) | 382 (34%) |
|  | *West* | 17 (27%) | 11 (27%) | 240 (24%) | 268 (24%) |
| Metropolitan area | *Yes* | 53 (84%) | 37 (90%) | 865 (85%) | 955 (85%) |

**Supplemental Table 2: Attitudes towards IGG**

| **Statement** | | **Percent** | |
| --- | --- | --- | --- |
|  |  | n=1,123 | |
| **IGG should be used to solve crimes^2,3^** | |  | |
| Always | | 20% | |
| Usually | | 22% | |
| Sometimes | | 37% | |
| Rarely | | 9% | |
| Never | | 5% | |
| Not sure | | 8% | |
| **IGG should be used to identify unidentified deceased individuals^2,3^** | |  | |
| Always | | 50% | |
| Usually | | 28% | |
| Sometimes | | 14% | |
| Rarely | | 3% | |
| Never | | 1% | |
| Not sure | | 5% | |
| **IGG should be used to identify deceased, abandoned infants^2^** | |  | |
| Always | | 55% | |
| Usually | | 22% | |
| Sometimes | | 12% | |
| Rarely | | 3% | |
| Never | | 2% | |
| Not sure | | 6% | |
| **IGG should be regulated** | |  | |
| Yes | | 78% | |
| Not sure | | 18% | |
| **IGG should be regulated by:^4^** | |  | |
| **(select all that apply)** | |  |  |
|  | Federal government | 54% | |
|  | State government | 40% | |
|  | County/city government | 21% | |
|  | Police leadership | 14% | |
|  | Other | 4% | |
| **Should be permitted to build family trees^2^:** | |  | |
| **(select all that apply)** | |  |  |
|  | The police | 24% | |
|  | Professional/independent genealogists | 67% | |
|  | Other law enforcement | 23% | |
|  | Not sure | 19% | |
| ^2^ Data available for main sample only (n=1,019) | |  | |
| ^3^ Percentages do not add to 100% due to rounding | | |  |
| ^4^ Among those who did not oppose regulation of IGG (96% answering “Yes” or “Not sure” combined, *n* = 1080) | | | |

**Supplemental Table 3: Attribute agreement**

| **Type of crime:** |  |
| --- | --- |
| Should there be limits on which crimes IGG can be used to investigate? |  |
| Yes | 71% |
| **Cases solved:** |  |
| Should the number of unsolved cases that can be solved using IGG play a role in whether the police are allowed to use IGG? |  |
| Yes | 63% |
| **Customer privacy:** |  |
| Should there be limits on the information about database customers the police can access when they use IGG? |  |
| Yes | 79% |
| **Family privacy:** |  |
| Should there be limits on how the police get DNA for IGG from relatives who are not genetic genealogy customers? |  |
| Yes | 82% |
| **Type of case:** |  |
| Should there be limits on when the police can use IGG in an investigation? |  |
| Yes | 68% |
| **Priority populations:** |  |
| Should the police prioritize cases involving different populations? |  |
| Yes | 47% |
|  |  |
