# Supplementary material for: Public preferences for investigative genetic genealogy (IGG) programs: A discrete choice experiment
Source: Genet Med Open. 2026 Sep 30;4:104499. doi: 10.1016/j.gimo.2026.104499 (PMC13743379; doi:10.1016/j.gimo.2026.104499)
Supplement: Supplementary file 3 [file mmc2.docx]

**Survey Questions***

**ForenSeq Genealogy DCE Survey**

Conducted for: Baylor College of Medicine

Conducted by: NORC at the University of Chicago

Sample Source: AmeriSpeak Probability-Based Panel

Sampled Population: General Population Age 18+

Date Fielded: 5/22/2023 – 6/12/2023

WINTRO_1.

Thank you for agreeing to participate in our new AmeriSpeak survey!

This survey is about police work.

As always, your answers are confidential.

CONSENT.

Personal Information and the Police

We want to know your opinions on how the police use different types of personal information to solve investigations. Your personal information may help police to identify suspected criminals more quickly and accurately. However, when they access and use your personal information, there are concerns that the police might intrude on your personal privacy or the privacy of your relatives. In this survey, we will ask for your opinions on police access and use of personal information. There are no right or wrong answers.

The survey will take about 20 minutes. Your participation is voluntary, and you may end your participation at any time. Your refusal to participate will involve no penalty or loss of benefits to which you might otherwise be entitled. We will ask you questions about you and your family. Some of these questions might make you uncomfortable or prompt negative emotions.

You must be at least 18 years old to participate. If you are willing to participate, please click the “Continue” button below. Proceeding with the survey will constitute your consent to participate.

REDIRECT.

This survey is hosted on one of our trusted partner websites. Once you complete the survey, you will be automatically redirected back to AmeriSpeak!

**If you are completing this survey on a mobile phone, we recommend turning your phone horizontally.**

Throughout the survey, there will be some questions that you will be required to answer. You will not be able to skip these questions.

Please click the “Continue” button below to be redirected to the main survey.

DISPLAY_GENETICS.

We will define some key terms before we look at the next group of questions.

**Genetics** is the study of DNA. You get DNA from your parents and pass it on to your children. You can learn about your genetics from DNA tests.

**Genealogy** is the study of families and how family members are related. Genealogy involves reviewing historical records and other documents to build your **family tree**. Your family tree includes many relatives. Some are close relatives, like your parents and siblings. Others are more distant relatives, like your great-grandparents and their cousins.

**Genetic genealogy** uses DNA test results to help build your family tree. If you get your DNA tested by a genetic genealogy company, the company can search its **database of customers** to find people who share DNA with you. These people are your **genetic relatives**. They have consented for the company to include their DNA test results in its database. They have also consented for the company to identify them as the genetic relatives of other customers.

GENE3.

**Genetic genealogy can be used to help build family trees.**

RESPONSE OPTIONS

1. True
2. False

[SHOW IF GENE3=1]

GENE3_CORRECT.

Correct, this is true.

[SHOW IF GENE3=2]

GENE3_INCORRECT.

That is incorrect. This is a true statement. Genetic genealogy involves using DNA test results to help build family trees.

GENE1.

**Have you ever participated in genetic genealogy?** Participating in genetic genealogy means you have used DNA test results to help build your family tree.

RESPONSE OPTIONS

1. Yes
2. No
77. Not sure

[SHOW IF GENE1=2,77]

GENE2.

**Are you interested in participating in genetic genealogy?**

RESPONSE OPTIONS

1. Yes
2. No
77. Not sure

IGG1.

The police can use genetic genealogy to help solve crime. This is called **investigative genetic genealogy** (or **‘IGG’** for short). IGG gives police leads on who might have committed a crime.

IGG1A.

IGG has the following basic steps:

1. The police collect DNA at the crime scene that was left by the person who committed the crime. That person is the **perpetrator**.

2. The police test the DNA and submit the DNA test results to a genetic genealogy company.

3. The company searches its database of customers and provides the police the names of the perpetrator’s genetic relatives and their predicted relationships to the perpetrator based on shared DNA.

4. The police use this information, as well as information from historical records and other documents, to build the perpetrator’s family tree.

5. The police look at the people on the family tree for leads on who might have committed the crime.

6. Sometimes the police test the DNA of specific people on the family tree who might not be database customers. This helps to narrow the investigation.

7. When **suspect leads** are identified, the police investigate them using regular police methods.

IGG1C.

IGG can help the police solve crimes that might not have been solved using regular police methods. IGG can also be used to exonerate people who were convicted for crimes they did not commit. However, there are concerns that IGG might intrude on the privacy of genetic genealogy database customers or their genetic relatives.

IGG1D.

**Had you heard of IGG before taking this survey?**

RESPONSE OPTIONS

1. Yes
2. Maybe, it sounds familiar
3. No

IGG2.

**The police get the DNA test results of database customers when they use IGG.**

RESPONSE OPTIONS

1. True
2. False

[SHOW IF IGG2=2]

IGG2_CORRECT.

Correct, this is false. When they use IGG, the police do not get customers’ DNA test results. Instead, the police are told which customers are genetically related to the perpetrator and how they may be related.

[SHOW IF IGG2=1]

IGG2_INCORRECT.

That is incorrect. This is a false statement. When they use IGG, the police do not get customers’ DNA test results. Instead, the police are told which customers are genetically related to the perpetrator and how they may be related.

IGG3.

**IGG tells the police who committed a crime.**

RESPONSE OPTIONS

1. True
2. False

[SHOW IF IGG3=2]

IGG3_CORRECT.

Correct, this is false. IGG only gives the police possible leads on suspects. The police then investigate suspect leads using regular police methods.

[SHOW IF IGG3=1]

IGG3_INCORRECT.

No, this is a false statement. IGG only gives the police possible leads on suspects. The police then investigate suspect leads using regular police methods.

IGG4.

**Do you believe the police should be allowed to use IGG to investigate a crime?**

RESPONSE OPTIONS

1. Always
2. Usually
3. Sometimes
4. Rarely
5. Never
77. Not sure

IGG5.

The police can use IGG to identify a deceased person’s body if they are not able to determine who the person is.

**Should the police be allowed to use IGG to identify a deceased person’s body?**

RESPONSE OPTIONS

1. Always
2. Usually
3. Sometimes
4. Rarely
5. Never
77. Not sure

IGG6.

The police can use IGG to identify babies who were abandoned shortly after they were born and died as a result. The baby’s parents might later be charged with the baby’s death many years later.

**Should the police be allowed to use IGG to identify the body of a deceased, abandoned baby?**

RESPONSE OPTIONS

1. Always
2. Usually
3. Sometimes
4. Rarely
5. Never
77. Not sure

IGG7.

**Do you believe that IGG should be regulated?** Regulating IGG means adopting laws or rules that must be followed when IGG is used.

RESPONSE OPTIONS

1. Yes
2. No
77. Not sure

IGG8.

**Who should regulate IGG?** Please select all that apply.

RESPONSE OPTIONS

1. The federal government
2. State governments
3. County or city governments
4. Police leadership
5. Other (please explain): [TEXTBOX]
77. Not sure

IGG9.

It is common for the police to work with independent genetic genealogists to build family trees for IGG. However, sometimes the police, or other people who work in law enforcement, build family trees.

**Who should be allowed to build family trees for IGG?** Please select all that apply.

RESPONSE OPTIONS

1. The police
2. Other people who work in law enforcement
3. Independent genetic genealogists
4. Other (please explain): [TEXTBOX]
77. Not sure

IGG10.

**Do you have any other ideas about how IGG should be used and/or regulated?**

[MEDIUM TEXTBOX]

IGG_PROG.

We will now ask you to consider different ways the police might use IGG. We will call these **IGG programs**. Each program is composed of possible benefits, possible harms, and process factors.

They are:

| **Possible benefits** | Which crimes |
| --- | --- |
|  | Solving additional cases |
| **Possible harms** | Privacy of database customers |
|  | Privacy of relatives who are not database customers |
| **Process factors** | When to use |
|  | Prioritizing populations |

Each of these possible benefits, possible harms, and process factors has different levels. We will present these levels on the next pages. We will use color and shading to help visualize the different levels. Some levels may not reflect current or future police activities.

IGG_CRIME.

**Which crimes**

IGG can be used to investigate different types of crime. Given concerns that IGG might intrude on some people’s privacy, IGG programs may limit the types of crimes it is used to investigate. We will consider programs where IGG is:

| **Used for any crime** | The police can use IGG to investigate any type of crime, including violent and non-violent crime |
| --- | --- |
| **Used for violent crime only** | The police can use IGG to investigate violent crime only |
| **Used for serial murder and rape only** | The police can use IGG to investigate serial murder and serial rape only |

**Should there be limits on which crimes IGG can be used to investigate?**

RESPONSE OPTIONS

1. Yes
2. No

IGG_ADD.

**Solving additional cases**

Some crimes that have not been solved using regular police methods can be solved using IGG. We will consider programs in which IGG:

| **Solves** **25% of unsolved cases** | IGG solves an additional 25 out of 100 crimes not solved using regular police methods |
| --- | --- |
| **Solves** **50% of unsolved cases** | IGG solves an additional 50 out of 100 crimes not solved using regular police methods |
| **Solves** **75% of unsolved cases** | IGG solves an additional 75 out of 100 crimes not solved using regular police methods |

**Should the number of unsolved cases that can be solved using IGG play a role in whether the police are allowed to use IGG?**

RESPONSE OPTIONS

1. Yes
2. No

IGG_PRIV_CUST.

**Privacy of database customers**

When they use IGG, the police submit the perpetrator’s DNA test results to a genetic genealogy company. The company searches its database of customers for the perpetrator’s genetic relatives. To protect customer privacy, IGG programs may limit the customer information the police can access. We will consider programs where the police are provided:

| **Access to customer names** | The police get only the names of customers related to the perpetrator |
| --- | --- |
| **Access to customer names and relationships** | The police get the names and possible relationships of customers related to the perpetrator |
| **Access to customer names, relationships, DNA tests** | The police get the names, possible relationships, and DNA test results of customers related to the perpetrator |

**Should there be limits on the information about database customers the police can access when they use IGG?**

RESPONSE OPTIONS

1. Yes
2. No

IGG_PRIV_REL.

**Privacy of relatives who are not database customers**

IGG involves building the perpetrator’s family tree. Many people on the tree are not genetic genealogy database customers. Sometimes, the police obtain DNA from a genetic relative on the family tree to help narrow the investigation. Limits can be placed on how the police get DNA from relatives for this purpose. We will consider programs where the police:

| **Collect relatives’ DNA voluntarily** | The police get DNA from relatives only if they voluntarily provide it |
| --- | --- |
| **Collect relatives’ DNA by warrant** | The police get DNA from relatives only if the police have a warrant to do so |
| **Collect relatives’ DNA secretly** | The police get DNA from relatives without them knowing, for example, by collecting DNA from something they threw in the trash, like a soda can |

**Should there be limits on how the police get DNA for IGG from relatives who are not database customers?**

RESPONSE OPTIONS

1. Yes
2. No

IGG_USE.

**When to use**

IGG is one of many methods the police might use in an investigation. The longer the police wait, the more likely they are to try other methods before IGG. Limits can be placed on when IGG can be used depending on the age and status of the case. We will consider programs where IGG is:

| **Used for cold cases** | The police can use IGG to investigate cases that were not solved using regular police methods |
| --- | --- |
| **Used for active cases** | The police can use IGG to investigate cases that are also being investigated using regular police methods |
| **Used for new cases** | The police can use IGG to investigate cases before trying regular police methods |

**Should there be limits on when the police can use IGG in an investigation?**

RESPONSE OPTIONS

1. Yes
2. No

IGG_PRIORITY.

**Prioritizing populations**

IGG programs may receive funding to address past injustices. This funding can be used to prioritize cases involving different populations of people. We will consider programs where IGG use:

| **Prioritizes women victims** | The police prioritize using IGG to investigate crimes against women |
| --- | --- |
| **Prioritizes minority victims** | The police prioritize using IGG to investigate crimes against racial/ethnic minorities |
| **Prioritizes wrongly convicted persons** | The police prioritize using IGG to investigate crimes involving people who may have been wrongly convicted |

**Should police use of IGG prioritize cases involving specific populations?**

RESPONSE OPTIONS

1. Yes
2. No

DISPLAY_IGG_PROG.

We will now ask you to compare IGG programs that vary in their possible benefits, possible harms, and process factors and indicate which IGG program you prefer. These programs may not reflect current or future police activities or policies. They were developed for this survey to explore what tradeoffs you would be willing to make.

We will begin with an example task. You will then be presented with 9 similar tasks.

If you are completing this survey on a mobile phone, we recommend turning your phone horizontally to view the tasks.

RANDOMIZE HALF OF RESPONDENTS TO SEE EXAMPLE_IGG1 AND HALF OF RESPONDENTS TO SEE EXAMPLE_IGG2.

[SHOW TO HALF OF RESPONDENTS]

EXAMPLE_IGG1.

Jordan is asked to complete the following task by indicating which IGG program is better for society.

**Suppose that you are asked to give input on which IGG program is better for society. You are presented with the following options. If these programs were the only options available, which would you choose?**

| **Program A** |  | **Program B** |  | **Program C** |
| --- | --- | --- | --- | --- |
| Used for serial murder and rape only |  | Used for any crime |  | IGG cannot be used at all |
| Solves 25% of unsolved cases |  | Solves 75% of unsolved cases |  |  |
| Access to customer names and relationships |  | Access to customer names, relationships, DNA tests |  |  |
| Collect relatives’ DNA by warrant |  | Collect relatives’ DNA voluntarily |  |  |
| Used for cold cases |  | Used for new cases |  |  |
| Prioritizes women victims |  | Prioritizes minority victims |  |  |
| - I would choose Program A |  | - I would choose Program B |  | - I would choose Program C |

Jordan considers Programs A and B. In Program A, IGG can only be used to investigate serial murder and rape and it solves an additional 25% of cases not solved using regular police methods. The police can access the names and relationships of database customers. They also can collect DNA from relatives who are not database customers with a warrant. Finally, Program A allows the police to use IGG to investigate cold cases and prioritizes cases involving women victims.

In Program B, IGG can be used to solve any crime and it solves an additional 75% of cases not solved using regular police methods. The police can access the names, relationship, and DNA tests of database customers. They also can collect DNA from relatives who are not database customers if they voluntarily provide their DNA. Finally, Program B allows the police to use IGG to investigate new cases and prioritizes cases involving minority victims.

Jordan likes some aspects of Program A and some aspects of Program B. Overall Jordan decides that Program A is better for society. Jordan also considered Program C but does not think that option is better.

[SHOW TO HALF OF RESPONDENTS]

EXAMPLE_IGG2.

Jordan is asked to complete the following task by indicating which IGG program is better for society.

**Suppose that you are asked to give input on which IGG program is better for society. You are presented with the following options. If these programs were the only options available, which would you choose?**

| **Program A** |  | **Program B** |  | **Program C** |
| --- | --- | --- | --- | --- |
| Used for serial murder and rape only |  | Used for any crime |  | IGG cannot be used at all |
| Solves 25% of unsolved cases |  | Solves 75% of unsolved cases |  |  |
| Access to customer names and relationships |  | Access to customer names, relationships, DNA tests |  |  |
| Collect relatives’ DNA by warrant |  | Collect relatives’ DNA voluntarily |  |  |
| Used for cold cases |  | Used for new cases |  |  |
| Prioritizes women victims |  | Prioritizes minority victims |  |  |
| - I would choose Program A |  | - I would choose Program B |  | - I would choose Program C |

Jordan considers Programs A and B. In Program A, IGG can only be used to investigate serial murder and rape and it solves an additional 25% of cases not solved using regular police methods. The police can access the names and relationships of database customers. They also can collect DNA from relatives who are not database customers with a warrant. Finally, Program A allows the police to use IGG to investigate cold cases and prioritizes cases involving women victims.

In Program B, IGG can be used to solve any crime and it solves an additional 75% of cases not solved using regular police methods. The police can access the names, relationship, and DNA tests of database customers. They also can collect DNA from relatives who are not database customers if they voluntarily provide their DNA. Finally, Program B allows the police to use IGG to investigate new cases and prioritizes cases involving minority victims.

Jordan likes some aspects of Program A and some aspects of Program B. Overall Jordan decides that Program B is better for society. Jordan also considered Program C but does not think that option is better.

PROGRAMMING NOTE: PLEASE USE EXPERIMENTAL DESIGN BELOW.

RANDOMIZE EACH RESPONDENT TO ONE BLOCK AND RECORD BLOCK.

WITHIN EACH BLOCK RANDOMIZE AND RECORD ORDER OF EACH TASK.

WITHIN EACH TASK, RANDOMIZE AND RECORD PROFILE A AND B TO LEFT POSITION OR MIDDLE POSITION. PROFILES A AND B SHOULD ALWAYS HAVE 6 TASKS. “NO IGG” PROFILE SHOULD ALWAYS APPEAR AT RIGHT POSITION.

1a or 2a=1 “Used for any crime”

1a or 2a=2 “Used for violent crime only”

1a or 2a=3 “Used for serial murder and rape only”

1b or 2b=1 “Solve 25% of unsolved cases”

1b or 2b=2 “Solve 50% of unsolved cases”

1b or 2b=3 “Solve 75% of unsolved cases”

1c or 2c=1 “Access to customer names”

1c or 2c=2 “Access to customer names and relationships”

1c or 2c=3 “Access to customer names, relationships, DNA tests”

1d or 2d=1 “Collect relatives’ DNA voluntarily”

1d or 2d=2 “Collect relatives’ DNA by warrant”

1d or 2d=3 “Collect relatives’ DNA secretly”

1e or 2e=1 “Used for cold cases”

1e or 2e=2 “Used for active cases”

1e or 2e=3 “Used for new cases”

1f or 2f=1 “Prioritizes women victims”

1f or 2f=2 “Prioritizes minority victims”

1f or 2f=3 “Prioritizes wrongly convicted persons”

| Block | Task | alt1a | alt1b | alt1c | alt1d | alt1e | alt1f | alt2a | alt2b | alt2c | alt2d | alt2.e | alt2.f |
| --- | --- | --- | --- | --- | --- | --- | --- | --- | --- | --- | --- | --- | --- |
| 1 | 1 | 3 | 3 | 2 | 1 | 3 | 2 | 1 | 2 | 3 | 3 | 2 | 1 |
| 1 | 2 | 1 | 3 | 2 | 3 | 2 | 3 | 3 | 1 | 1 | 2 | 3 | 2 |
| 1 | 3 | 3 | 3 | 2 | 1 | 2 | 2 | 1 | 1 | 1 | 3 | 1 | 1 |
| 1 | 4 | 1 | 3 | 1 | 2 | 3 | 1 | 3 | 1 | 2 | 3 | 1 | 3 |
| 1 | 5 | 2 | 2 | 2 | 2 | 1 | 2 | 3 | 3 | 3 | 1 | 3 | 1 |
| 1 | 6 | 3 | 2 | 2 | 3 | 1 | 1 | 2 | 1 | 3 | 1 | 2 | 2 |
| 1 | 7 | 3 | 1 | 2 | 2 | 3 | 3 | 2 | 2 | 3 | 1 | 1 | 2 |
| 1 | 8 | 1 | 1 | 1 | 1 | 1 | 2 | 2 | 2 | 2 | 3 | 3 | 3 |
| 1 | 9 | 2 | 1 | 3 | 2 | 3 | 1 | 1 | 3 | 1 | 1 | 2 | 3 |
| 2 | 10 | 2 | 3 | 3 | 2 | 2 | 3 | 3 | 2 | 2 | 1 | 3 | 2 |
| 2 | 11 | 3 | 2 | 3 | 3 | 1 | 2 | 2 | 1 | 2 | 2 | 2 | 3 |
| 2 | 12 | 1 | 2 | 3 | 2 | 3 | 3 | 2 | 3 | 1 | 3 | 2 | 2 |
| 2 | 13 | 1 | 1 | 1 | 3 | 2 | 2 | 2 | 2 | 2 | 1 | 1 | 1 |
| 2 | 14 | 1 | 2 | 2 | 1 | 2 | 1 | 2 | 3 | 1 | 3 | 1 | 3 |
| 2 | 15 | 1 | 1 | 1 | 3 | 1 | 1 | 3 | 2 | 3 | 2 | 2 | 2 |
| 2 | 16 | 3 | 1 | 3 | 3 | 1 | 2 | 2 | 2 | 1 | 1 | 3 | 1 |
| 2 | 17 | 3 | 3 | 1 | 2 | 1 | 3 | 1 | 1 | 2 | 3 | 2 | 2 |
| 2 | 18 | 1 | 1 | 3 | 1 | 3 | 3 | 3 | 3 | 1 | 2 | 2 | 1 |
| 3 | 19 | 3 | 2 | 1 | 1 | 2 | 1 | 1 | 3 | 2 | 2 | 1 | 3 |
| 3 | 20 | 2 | 1 | 1 | 1 | 3 | 3 | 1 | 3 | 3 | 2 | 1 | 1 |
| 3 | 21 | 2 | 3 | 3 | 1 | 1 | 1 | 3 | 2 | 1 | 3 | 3 | 3 |
| 3 | 22 | 1 | 2 | 1 | 2 | 2 | 3 | 2 | 1 | 3 | 3 | 3 | 1 |
| 3 | 23 | 3 | 3 | 3 | 3 | 2 | 1 | 1 | 2 | 1 | 2 | 1 | 2 |
| 3 | 24 | 2 | 3 | 2 | 3 | 3 | 2 | 3 | 1 | 3 | 2 | 2 | 3 |
| 3 | 25 | 2 | 2 | 1 | 1 | 1 | 3 | 1 | 3 | 2 | 2 | 3 | 2 |
| 3 | 26 | 2 | 1 | 2 | 2 | 2 | 1 | 1 | 3 | 3 | 1 | 3 | 3 |
| 3 | 27 | 2 | 2 | 3 | 3 | 3 | 2 | 3 | 1 | 2 | 1 | 1 | 1 |

IGG_TASK1.

**Suppose that you are asked to give input on which IGG program is better for society. You are presented with the following options. If these programs were the only options available, which would you choose?**

| **Program A** |  | **Program B** |  | **Program C** |
| --- | --- | --- | --- | --- |
| Used for serial murder and rape only |  | Used for any crime |  | IGG cannot be used at all |
| Solves 75% of unsolved cases |  | Solves 50% of unsolved cases |  |  |
| Access to customer names and relationships |  | Access to customer names, relationships, DNA tests |  |  |
| Collect relatives’ DNA voluntarily |  | Collect relatives’ DNA secretly |  |  |
| Used for new cases |  | Used for active cases |  |  |
| Prioritizes minority victims |  | Prioritizes women victims |  |  |
| - I would choose Program A |  | - I would choose Program B |  | - I would choose Program C |

REPEAT FORMATTING FOR IGG_TASK2 THROUGH IGG_TASK9, USING EXPERIMENTAL DESIGN AS INDICATED ABOVE.

IGG_TASK_DEBRIEF.

**What did you think?**

These statements refer to the tasks that you just completed. Please mark your answers in the grid.

GRID ITEMS

A. The questions were easy to understand
B. The questions were easy to answer
C. My answers showed my real preferences
D. The questions were relevant to me

RESPONSE OPTIONS

1. Strongly disagree
2. Disagree
3. Neither agree nor disagree
4. Agree
5. Strongly agree

IGG_DEBRIEF_OE.

**Was there anything about the presentation of these tasks that made them difficult to understand or answer?**

[MEDIUM TEXTBOX]

EMP1.

**Have you ever worked in law enforcement?** Examples of law enforcement jobs include police officer, detective, sheriff, crime analyst, and parole officer.

RESPONSE OPTIONS

1. Yes
2. No

EMP2.

**Have you ever worked in the legal field?** Examples of legal field jobs include lawyer, paralegal, and judge.

RESPONSE OPTIONS

1. Yes
2. No

EMP3.

**What is your general view of the police?**

RESPONSE OPTIONS

1. Very positive
2. Somewhat positive
3. Neither positive nor negative
4. Somewhat negative
5. Very negative

EXP1.

**Have you ever been arrested, charged with, or convicted of a crime?**

RESPONSE OPTIONS

1. Yes, violent crime only
2. Yes, non-violent crime only
3. Yes, both violent and non-violent crime
4. No
99. Prefer not to answer

EXP2.

**To your knowledge, has anyone close to you ever been arrested, charged with, or convicted of a crime?**

RESPONSE OPTIONS

1. Yes, violent crime only
2. Yes, non-violent crime only
3. Yes, both violent and non-violent crime
4. No
99. Prefer not to answer

EXP3.

**Have you ever been the victim of a crime?**

RESPONSE OPTIONS

1. Yes, violent crime only
2. Yes, non-violent crime only
3. Yes, both violent and non-violent crime
4. No
99. Prefer not to answer

EXP4.

**To your knowledge, has anyone close to you ever been the victim of crime?**

RESPONSE OPTIONS

1. Yes, violent crime only
2. Yes, non-violent crime only
3. Yes, both violent and non-violent crime
4. No
99. Prefer not to answer

EXP5.

**Please select the best description of your neighborhood.**

RESPONSE OPTIONS

1. High crime
2. Moderate crime
3. Low crime
77. I don’t know
99. Prefer not to answer

SELF.

**Please answer a few final questions about yourself.**

GRID ITEMS

A. I am usually optimistic about my future.
B. I have a lot of self-control.
C. I am actively working to improve my health.
D. I am a risk taker.
E. I am good with numbers.

RESPONSE OPTIONS

1. Strongly disagree
2. Disagree
3. Neither agree nor disagree
4. Agree
5. Strongly agree

POLICE_FINAL.

**Is there anything else you would like to tell us about your views of the police and IGG?**

[MEDIUM TEXTBOX]

SURV_COMP.

Thank you for finishing the survey.

Please select the “Next” arrow to complete the survey.

**Demographic Profile:**

**Additional questions asked of panelists prior to this survey**

**and are included with the survey data**

| Variable | Values |
| --- | --- |
| Gender | 1 = Male |
|  | 2 = Female |
| Age | Age in years |
| Age (7 categories) | 1 = 18-24; 2 = 25-34; 3 = 35-44; 4 = 45-54; 5 = 55-64; 6 = 65-74; 7 = 75+ |
| Age (4 categories) | 1 = 18-29; 2 = 30-44; 3 = 45-59; 4 = 60+ |
| Education (5 categories) | 1 = Less than HS |
|  | 2 = HS graduate |
|  | 3 = Vocational/tech school/some college/associates |
|  | 4 = Bachelor’s degree |
|  | 5 = Post grad study/professional degree |
| Race/Ethnicity | 1 = White, Non-Hispanic |
|  | 2 = Black, Non-Hispanic |
|  | 3 = Other, Non-Hispanic |
|  | 4 = Hispanic |
|  | 5 = 2+ races, Non-Hispanic |
|  | 6 = Asian/Pacific Islander, Non-Hispanic |
| Housing Type | 1 = A one-family house detached from any other house |
|  | 2 = A one-family house attached to one or more houses |
|  | 3 = A building with 2 or more apartments |
|  | 4 = A mobile home or trailer |
|  | 5 = Boat, RV, van, etc. |
| Household Income (18 categories) | 1 = Less than $5,000 2 = $5,000 to $9,999 |
|  | 3 = $10,000 to $14,999 4 = $15,000 to $19,999 |
|  | 5 = $20,000 to $24,999 6 = $25,000 to $29,999 |
|  | 7 = $30,000 to $34,999 8 = $35,000 to $39,999 |
|  | 9 = $40,000 to $49,999 10 = $50,000 to $59,999 |
|  | 11 = $60,000 to $74,999 12 = $75,000 to $84,999 |
|  | 13 = $85,000 to $99,999 14 = $100,000 to $124,999 |
|  | 15 = $125,000 to $149,999 16 = $150,000 to $174,999 |
|  | 17 = $175,000 to $199,999 18 = $200,000 or more |
| Household Income (9 categories) | 1 = Less than $10,000 |
|  | 2 = $10,000 to $19,999 |
|  | 3 = $20,000 to $29,999 |
|  | 4 = $30,000 to $39,999 |
|  | 5 = $40,000 to $49,999 |
|  | 6 = $50,000 to $74,999 |
|  | 7 = $75,000 to $99,999 |
|  | 8 = $100,000 to $149,999 |
|  | 9 = $150,000 or more |
| Household Income (4 categories) | 1 = Less than $30,000 |
|  | 2 = $30,000 to $59,999 |
|  | 3 = $60,000 to $99,999 |
|  | 4 = $100,000 or more |
| Marital Status | 1 = Married |
|  | 2 = Widowed |
|  | 3 = Divorced |
|  | 4 = Separated |
|  | 5 = Never married |
|  | 6 = Living with partner |
| Metropolitan Statistical Area  Status | 0 = Non-Metro |
|  | 1 = Metro (as defined US OMB Core-Based Statistical Area) |
| Home Internet Access | 0 = No |
|  | 1 = Yes |
| Telephone Service | 1 = Landline telephone only |
|  | 2 = Have a landline, but mostly use cellphone |
|  | 3 = Have cellphone, but mostly use landline |
|  | 4 = Cellphone only |
|  | 5 = No telephone service |
| Ownership of Living Quarters | 1 = Owned or being bought by you or someone in your household |
|  | 2 = Rented for cash |
|  | 3 = Occupied without payment of cash rent |
| Region 4 (US Census) | 1 = Northeast |
|  | 2 = Midwest |
|  | 3 = South |
|  | 4 = West |
| Region 9 (US Census) | 1 = New England |
|  | 2 = Mid-Atlantic |
|  | 3 = East-North Central |
|  | 4 = West-North Central |
|  | 5 = South Atlantic |
|  | 6 = East-South Central |
|  | 7 = West-South Central |
|  | 8 = Mountain |
|  | 9 = Pacific |
| State | State of residence |
| Household Size | Total number of members in household |
| HH members, age 0-1 | Number of household members in age group |
| HH members, age 2-5 | Number of household members in age group |
| HH members, age 6-12 | Number of household members in age group |
| HH members, age 13-17 | Number of household members in age group |
| HH members, age 18+ | Number of household members in age group |
| Current Employment Status | 1 = Working - as a paid employee |
|  | 2 = Working - self-employed |
|  | 3 = Not working - on temporary layoff from a job |
|  | 4 = Not working - looking for work |
|  | 5 = Not working – retired |
|  | 6 = Not working – disabled |
|  | 7 = Not working – other |
